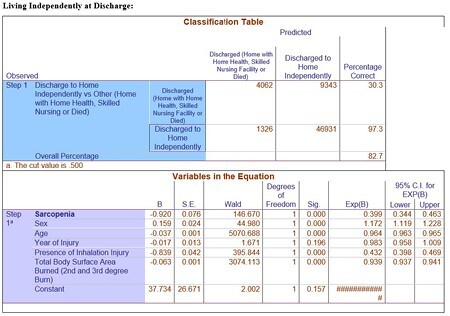# 61 The Influence of Sarcopenia on Patient Outcomes Among Burn Patients: A National Burn Repository Study

**DOI:** 10.1093/jbcr/irae036.053

**Published:** 2024-04-17

**Authors:** Elizabeth Blears, Jessica Ballou, Andrew Murton, Julie A Caffrey

**Affiliations:** Tower Health, Wyomissing, Pennsylvania; Johns Hopkins, Baltimore, Maryland; University of Texas-Medical Branch, Galveston, Texas; Johns Hopkins University School of Medicine, Baltimore, MD; Tower Health, Wyomissing, Pennsylvania; Johns Hopkins, Baltimore, Maryland; University of Texas-Medical Branch, Galveston, Texas; Johns Hopkins University School of Medicine, Baltimore, MD; Tower Health, Wyomissing, Pennsylvania; Johns Hopkins, Baltimore, Maryland; University of Texas-Medical Branch, Galveston, Texas; Johns Hopkins University School of Medicine, Baltimore, MD; Tower Health, Wyomissing, Pennsylvania; Johns Hopkins, Baltimore, Maryland; University of Texas-Medical Branch, Galveston, Texas; Johns Hopkins University School of Medicine, Baltimore, MD

## Abstract

**Introduction:**

Over recent decades, improvements in triage and hospital protocols have resulted in remarkable increases in survival following major burns. Despite this, patients continue to experience profound muscle cachexia, in large part, a consequence of the hypermetabolic response to burn injury. However, the rates of cachexia among burn and adult patients are not well established across multiple burn centers.

**Methods:**

The National Burn Repository (NBR) was used to explore the rate of sarcopenia and associated characteristics of patients diagnosed with sarcopenia while undergoing burn treatment in both the acute and follow-up setting. Diagnoses of sarcopenia was made according to appropriate ICD-9 and ICD-10 codes. Appropriate descriptive statistics were used to characterize the burden of muscle wasting and sarcopenia among burn patients with alpha < 0.05 as significant. Binomial and linear multivariate regression was performed to determine whether sarcopenia was an independent predictor of adverse burn outcomes after adjusting for common confounding variables, such as age, burn size and comorbidities.

**Results:**

In total, the NBR provided 84,438 adult and pediatric burn patients injured between 2000-2018 from over 100 burn centers predominantly in the United States. Only 2.5% (N=2,118) of the patients in the NBR were diagnosed with having sarcopenia or other forms of muscle atrophy. Patients with sarcopenia were significantly older (: 51.9 years (SD±21) vs 35.5 years (SD±23.6), P< 0.001), larger Total Body Surface Area burn (mean: 16.1% (SD±19.2) vs 7.1% (SD±11.5), P< 0.001) and required more surgical procedures (mean: 21.9 total procedures (SD±30.4) vs 7.9 total procedures (SD±13.7), P< 0.001). After adjusting for confounding variables, sarcopenia remained an independent predictor of ability to discharge to independent living (vs nursing facility or home with home nursing), longer inpatient days as well as increased number of surgical procedures (P < 0.001).

**Conclusions:**

Sarcopenia is an independent risk factor for adverse outcomes in burn patients, such as decreased likelihood of discharge to independent living, increased length of stay inpatient and increased number of surgical procedures. Confidence in these findings would be improved with more accurate data collection, as the diagnosis of sarcopenia is likely under-reported, under-diagnosed or both.

**Applicability of Research to Practice:**

Understanding the role of sarcopenia in clinical outcomes can better optimize nutrition and exercise service that can lead to better long-term outcomes.